# The Unrecognized Burden of Influenza in Young Kenyan Children, 2008-2012

**DOI:** 10.1371/journal.pone.0138272

**Published:** 2015-09-17

**Authors:** Meredith L. McMorrow, Gideon O. Emukule, Henry N. Njuguna, Godfrey Bigogo, Joel M. Montgomery, Bryan Nyawanda, Allan Audi, Robert F. Breiman, Mark A. Katz, Leonard Cosmas, Lilian W. Waiboci, Jazmin Duque, Marc-Alain Widdowson, Joshua A. Mott

**Affiliations:** 1 Influenza Division, National Center for Immunization and Respiratory Diseases, Centers for Disease Control and Prevention (CDC), Atlanta, Georgia, United States of America; 2 United States Public Health Service, Rockville, Maryland, United States of America; 3 Centers for Disease Control and Prevention-Kenya Country Office, Nairobi, Kenya; 4 Kenya Medical Research Institute (KEMRI), Nairobi and Kisumu, Kenya; 5 Division of Global Health Protection, Center for Global Health, Centers for Disease Control and Prevention, Atlanta, Georgia, United States of America; 6 Emory Global Health Institute, Emory University, Atlanta, Georgia, United States of America; 7 Battelle, Atlanta, Georgia, United States of America; National Institutes of Health, UNITED STATES

## Abstract

Influenza-associated disease burden among children in tropical sub-Saharan Africa is not well established, particularly outside of the 2009 pandemic period. We estimated the burden of influenza in children aged 0–4 years through population-based surveillance for influenza-like illness (ILI) and acute lower respiratory tract illness (ALRI). Household members meeting ILI or ALRI case definitions were referred to health facilities for evaluation and collection of nasopharyngeal and oropharyngeal swabs for influenza testing by real-time reverse transcription polymerase chain reaction. Estimates were adjusted for health-seeking behavior and those with ILI and ALRI who were not tested. During 2008–2012, there were 9,652 person-years of surveillance among children aged 0–4 years. The average adjusted rate of influenza-associated hospitalization was 4.3 (95% CI 3.0–6.0) per 1,000 person-years in children aged 0–4 years. Hospitalization rates were highest in the 0–5 month and 6–23 month age groups, at 7.6 (95% CI 3.2–18.2) and 8.4 (95% CI 5.4–13.0) per 1,000 person-years, respectively. The average adjusted rate of influenza-associated medically attended (inpatient or outpatient) ALRI in children aged 0–4 years was 17.4 (95% CI 14.2–19.7) per 1,000 person-years. Few children who had severe laboratory-confirmed influenza were clinically diagnosed with influenza by the treating clinician in the inpatient (0/33, 0%) or outpatient (1/109, 0.9%) settings. Influenza-associated hospitalization rates from 2008–2012 were 5–10 times higher than contemporaneous U.S. estimates. Many children with danger signs were not hospitalized; thus, influenza-associated severe disease rates in Kenyan children are likely higher than hospital-based estimates suggest.

## Introduction

The burden of influenza in children in the United States and other temperate countries is well-described.[1–8] Influenza-associated disease burden among children in tropical sub-Saharan Africa is not well established, particularly outside of the 2009 pandemic period. The epidemiology of influenza may be different in sub-Saharan Africa due to less predictable seasonality and the high prevalence of risk factors for severe influenza including HIV.[1, 9–11] In South Africa, HIV-infected persons experienced 4–8 times the age-adjusted incidence of severe influenza infection and 4 times the risk of influenza-associated death compared to HIV-uninfected persons.[12] Following the 2009 pandemic, a global pooled analysis found immune compromise was associated with a pooled relative risk of death of 27.7, but lacked sufficient information about HIV status to determine if a specific association existed.[13] In addition, a high case fatality risk has been reported from several influenza outbreaks in Africa. [14–17]

Among 14 African countries during 2006–2010, 10% of children 0–4 years old hospitalized with severe acute respiratory illness (SARI) were influenza-positive by real-time reverse transcription-polymerase chain reaction (rRT-PCR).[18] Hospital-based surveillance in Western Kenya identified influenza among 6.9% of inpatient and 13.8% of outpatient children aged 0–4 years [19] but few surveillance data have been used to assess rates of disease. A study of influenza-associated hospitalizations from Bondo District, reported an estimated 1.4 (95% CI: 1.2–1.7) hospitalizations per 1000 children.[20] However, this may also be an underestimate due to limited access to the study hospital and a lack of adjustment for health-seeking at other facilities. Other publications from Kenya included annual rates that were heavily influenced by inclusion of the pandemic period.[21–23] We estimated the burden of influenza in children aged 0–4 years through population-based influenza-like illness (ILI) and acute lower respiratory tract illness (ALRI) surveillance in 2 sites in Kenya, adjusting for the completeness of surveillance and health-seeking at other facilities. We also assessed risk factors for influenza infection and the likelihood of providers to clinically diagnose influenza.

## Materials and Methods

The Kenya Medical Research Institute (KEMRI) and the Centers for Disease Control-Kenya (CDC-Kenya) initiated population-based infectious disease surveillance in late 2005 in two sites in Kenya: Kibera, a large, informal urban settlement in Nairobi, and Lwak, a rural area in Western Kenya. All participating households provided written informed consent with parents/guardians providing written consent for participating children. The study was approved by the Institutional Review Board of the US Centers for Disease Control and Prevention (#4556) and the Ethical Review Committee of the Kenya Medical Research Institute (#932). The study sites and surveillance methods have been described previously.[21, 24, 25] Briefly, the Lwak population is approximately 25,000 in 33 villages within 5 kilometers of St. Elizabeth Lwak Mission Hospital. Located along Lake Victoria, malaria transmission is endemic. Kibera, the urban site with a surveillance population of approximately 28,000 in just 0.42km^2^, has semi-permanent housing with dirt paths and open sewers. Malaria is not endemic due to the high altitude. A home-based testing and counseling program in 2008 found HIV prevalence among adults was 14.9% in Kibera and 17.6% in Lwak. In both sites, participants must have resided in the area for at least 4 months prior to enrollment and can access free healthcare at centrally located clinics. There is no hospital-based surveillance in Kibera due to the large number of hospitals in Nairobi that may provide care to enrolled participants.

From January 2008 to December 2012, in both sites, we conducted household surveillance for ILI and ALRI every 2 weeks to capture all persons with ILI in the household regardless if they sought care or not. Household surveillance was increased to weekly from September 2009 to June 2011 following the emergence of the influenza A (H1N1)pdm09 strain. Household members with ILI or ALRI were referred to study clinics for evaluation, sample collection, and treatment. The ILI case definition was a measured temperature of ≥38°C plus cough or sore throat with onset in the last 14 days. The first 5 ILI cases presenting to the study clinic each day were sampled to test for influenza infection. The case definition for ALRI for children 0–4 years old was based on a modified World Health Organization (WHO) Integrated Management of Childhood Illness (IMCI) severe pneumonia definition: cough or difficulty breathing with presence of an IMCI danger sign or oxygen saturation ≤90% on room air. IMCI danger signs include inability to drink, vomiting everything, convulsions, lethargy or unconsciousness. ILI and/or ALRI reports that occurred within 7 days of each other were assumed to be the same ALRI episode. Both nasopharyngeal and oropharyngeal specimens, and clinical data were obtained from patients who met the ILI or ALRI case definition and attended the study outpatient clinic (both sites) or who met the ALRI case definition and were admitted to the study hospital in Lwak. Specimens were tested by rRT-PCR for influenza viruses at the CDC-Kenya Laboratory in Nairobi.[26]

We first compared characteristics (gender, age, weight-for-age, malaria co-infection, urban/rural residence, household size or economic quintile) of children who were tested for influenza with those who were not. Weight-for-age was assessed using standardized WHO growth charts and assigning a Z score for standard deviation above/below mean weight for age. Malaria co-infection was determined by blood smear or rapid diagnostic test. Residence in Kibera (urban) or Lwak (rural) serves as a proxy for urban/rural residence. Economic quintile was determined for residents of Lwak only based upon multiple correspondence analysis derived from household assets. These data were not available for the Kibera site.

We examined differences between children who tested positive for influenza with those who tested negative. Lastly we compared those children who met the ALRI case definition and were hospitalized with those who also met the ALRI case definition but were not hospitalized. Pearson’s chi-square tests and Wald chi-square tests using the logistic regression were used to assess differences in categorical variables, and Wilcoxon rank-sum tests to test for differences in median age. We also performed multivariable analysis for factors associated with influenza infection among ILI and ALRI patients (data not shown). Variables that had p-values <0.2 in the univariate analysis were included in multivariable analysis.

Population-based rates of influenza-associated hospitalization and medically attended ALRI (inpatient and outpatient) were calculated for children aged 0–5 months, 6–23 months, 24–59 months, and 0–4 years. Crude inpatient and outpatient rates were adjusted for health-seeking outside the study clinics by age group and year (as reported in home visits) and the proportion influenza-positive among tested children was applied to those meeting the case definition but from whom no specimen was collected (Appendix 1). Results are presented as events per 1000 person-years based on the population under surveillance. The 95% confidence intervals (CIs) were calculated using the Poisson approximation method. Statistical significance was set to p <0.05 in all analyses. Data analysis was performed using Stata version 12.1 (Stata Corp, College Station, Texas).

## Results

### Surveillance Summary

During 2008–2012, a total person-time of 9652 person-years follow-up was recorded among enrolled children aged 0–4 years. Recorded person-time varied minimally by month with the exception of December (p<0.001) (S1 Table). During 2008–2012, among enrolled children aged 0–4 years in the two study sites, 82,625 episodes of ILI were reported and 8493 (10.0%) episodes led to care in a study clinic. During the 5 years of household surveillance, 71% of children in Kibera and 58% of children in Lwak who were reported as receiving medical care for ILI were taken to a study clinic. Of the 8493 children with ILI who visited study clinics, 3128 (37.0%) were tested for influenza viruses and 358 (11.4%) tested positive. During the five years of surveillance in Lwak, 2566 children were reported hospitalized for all causes and 2340 (91%) of these were admitted to the study hospital. Of these 2340 children, 605 (25.9%) met the ALRI case definition, 242 (40.0%) were tested for influenza viruses of which 20 (8.3%) tested positive. Among these 2340 hospitalized children there were also 13 children with ILI or respiratory illness who were hospitalized and tested positive for influenza but did not meet the ALRI case definition. During the 5 years of surveillance, 74% of children in Kibera and 62% of children in Lwak who reported seeking care for ALRI came to a study clinic or hospital. Few children who had severe laboratory-confirmed influenza were clinically diagnosed with influenza by the treating clinician in the inpatient (0/33, 0%) or outpatient (1/109, 0.9%) settings despite a global pandemic occurring during the surveillance period. Among 33 children with influenza hospitalized in Lwak, clinicians gave a primary or secondary diagnosis of malaria (19, 58%), pneumonia or URTI (17, 52%), anemia (3, 9%), and other conditions (9, 27%). Among 109 influenza-positive outpatient children with ALRI, clinicians gave a primary or secondary diagnosis of pneumonia or URTI (81, 74%), malaria (15, 16%), diarrhea (9, 8.3%) or other conditions (37, 34%).

Among influenza-infected ALRI and ILI cases, the circulating virus strains changed over the surveillance period. Prior to the pandemic, influenza A(H3N2) (47%) and influenza B (31%) predominated among samples that were typed and subtyped. In 2009 and 2010, influenza A(H1N1)pdm09 was the predominant virus (56%). The pandemic strain (37%) co-circulated with influenza B (57%) in 2011; and in 2012 there was co-circulation of influenza A(H3N2) (50%) and B (44%) viruses.

### Comparison of Tested/Untested and Influenza Positive/Negative

Children with ILI who were tested for influenza were more likely to live in the rural area and have malaria co-infection compared to those who were not tested (p<0.001) (Table 1). Similarly, children with ALRI who were tested for influenza were more likely to live in the rural area (p = 0.025) and as likely to have malaria co-infection (p = 0.170) as those who were not tested.

**Table 1:** Characteristics of Children with Influenza-like Illness (ILI) and Acute Lower Respiratory Tract Illness (ALRI) without Laboratory Testing vs Children with Nasopharyngeal/Oropharyngeal (NP/OP) Sample Tested for Influenza, 2008–2012.

	Children with ILI				Children with ALRI			
	Total (N = 8,493)	No lab testing (N = 5,365)	NP/OP sample tested (N = 3,128)	p-value	Total (N = 3,578)	No lab testing (N = 2,516)	NP/OP sample tested (N = 1,062)	p-value
**Sex**								
Male, n(%)	4312(50.8)	2680(50.0)	1632(52.2)	0.050	1888(52.8)	1332(52.9)	556(52.4)	0.800
Female, n(%)	4181(49.2)	2685(50.0)	1496(47.8)	Ref	1690(47.2)	1184(47.1)	506(47.6)	Ref
**Age Group**								
0–5 months, n(%)	478(5.6)	299(5.6)	179(5.7)	0.910	494(13.8)	371(14.7)	123(11.6)	0.040
6–23 months, n(%)	3335(39.3)	2128(39.7)	1207(38.6)	0.320	1692(47.3)	1169(46.5)	523(49.2)	0.520
24–59 months, n(%)	4680(55.1)	2938(54.8)	1742(55.7)	Ref	1392(38.9)	976(38.8)	416(39.2)	Ref
Median age years (Range)	8493	2.2(0.0–4.9)	2.2(0.0–4.9)	0.510	3578	1.5(0.0–4.9)	1.5(0.0–4.9)	0.140
**Co-morbidity**								
Weight for age								
≥-2z scores, n(%)	7169(84.4)	4556(84.9)	2613(83.5)	Ref	2745(76.7)	1935(76.9)	810(76.3)	Ref
<-2z scores, n(%)	1324(15.6)	809(15.1)	515(16.5)	0.090	833(23.3)	581(23.1)	252(23.7)	0.745
Malaria testing*								
Negative	3351(39.5)	2212(41.2)	1139(36.4)	<0.001	1418(39.6)	888(35.3)	530(49.9)	0.170
Positive	4068(47.9)	2394(44.6)	1674(53.5)	Ref	702(19.6)	461(18.3)	241(22.7)	Ref
Not tested	1074(12.6)	759(14.1)	315(10.1)		1458(40.7)	1167(46.4)	291(27.4)	
Residence								
Urban (Kibera), n(%)	2906(34.2)	2253(42.0)	653(20.9)	<0.001	2322(64.9)	1662(66.1)	660(62.1)	0.025
Rural (Lwak), n(%)	5587(65.8)	3112(58.0)	2475(79.1)	Ref	1256(35.1)	854(33.9)	402(37.9)	Ref
Household size								
<5, n(%)	3102(36.5)	1990(37.1)	1112(35.5)	0.154	1499(41.9)	1056(42.0)	443(41.7)	0.862
≥5, n(%)	5391(63.5)	3375(62.9)	2016(64.5)	Ref	2079(58.1)	1460(58.0)	619(58.3)	Ref
Socioeconomic Status (Lwak only)								
1st quintile, n(%)	541(9.7)	262(8.4)	279(11.3)	Ref	119(9.5)	77(9.0)	42(10.4)	Ref
2nd quintile, n(%)	746(13.4)	376(12.1)	370(14.9)	0.490	171(13.6)	110(12.9)	61(15.2)	0.950
3rd quintile, n(%)	801(14.3)	363(11.7)	438(17.7)	0.260	210(16.7)	142(16.6)	68(16.9)	0.590
4th quintile, n(%)	934(16.7)	461(14.8)	473(19.1)	0.730	200(15.9)	128(15.0)	72(17.9)	0.830
5th quintile, n(%)	1062(19.0)	493(15.8)	569(23.0)	0.450	194(15.4)	123(14.4)	71(17.7)	0.820
Unknown, n(%)	1503(26.9)	1157(37.2)	346(14.0)	<0.001	362(28.8)	274(32.1)	88(21.9)	0.020

*chi-square test done only for those with malaria test

Among children with ILI who were tested for influenza, influenza-positive children were slightly older, and were less likely to have malaria co-infection (p<0.001) (Table 2). Children with ILI at the urban site were more likely to have influenza than those at the rural site (19.1% vs 9.4% influenza positive, p<0.001). Household size and wealth quintile were not associated with influenza infection (p>0.05). Similarly, children with ALRI who tested positive for influenza were less likely to have malaria co-infection than those who tested negative (p = 0.039). No statistically significant differences were observed between gender, age, weight-for-age, urban/rural residence, household size or socioeconomic quintile between influenza-positive and influenza-negative children with ALRI (Table 2).

**Table 2:** Characteristics of Children with Influenza-like Illness (ILI) or Acute Lower Respiratory Tract Illness (ALRI) by Influenza Test Result, 2008–2012.

	Children with ILI				Children with ALRI			
	Total (N = 3,128)	Influenza Positive (N = 358)	Influenza Negative (N = 2,770)	p-value	Total (N = 1,062)	Influenza Positive (N = 129)	Influenza Negative (N = 933)	p-value
**Sex**								
Male, n(%)	1632(52.2)	178(49.7)	1454(52.5)	0.320	556(52.4)	72(55.8)	484(51.9)	0.410
Female, n(%)	1496(47.8)	180(50.3)	1316(47.5)	Ref	506(47.6)	57(44.2)	449(48.1)	Ref
**Age Group**								
0–5 months, n(%)	179(5.7)	14(3.9)	165(6.0)	0.050	123(11.6)	9(7.0)	114(12.2)	0.100
6–23 months, n(%)	1207(38.6)	116(32.4)	1091(39.4)	<0.001	523(49.2)	67(51.9)	456(48.9)	0.910
24–59 months, n(%)	1742(55.7)	228(63.7)	1514(54.7)	Ref	416(39.2)	53(41.1)	363(38.9)	Ref
Median age years (Range)	3128	2.6(0.1–4.9)	2.2(0.1–4.9)	<0.001	1062	1.6(0.1–4.9)	1.5(0.0–4.9)	0.150
**Co-morbidity**								
Weight for age								
≥-2z scores, n(%)	2613(83.5)	296(82.7)	2317(83.6)	Ref	810(76.3)	97(75.2)	713(76.4)	Ref
<-2z scores, n(%)	515(16.5)	62(17.3)	453(16.4)	0.640	252(23.7)	32(24.8)	220(23.6)	0.740
Malaria testing*								
Negative	1140(36.4)	209(58.4)	931(33.6)	Ref	531(50.0)	77(59.7)	454(48.7)	Ref
Positive	1674(53.5)	107(29.9)	1567(56.6)	<0.001	241(22.7)	22(17.1)	219(23.5)	0.039
Not tested	314(10.0)	42(11.7)	272(9.8)		290(27.3)	30(23.3)	260(27.9)	
**Residence**								
Urban (Kibera), n(%)	653(20.9)	125(34.9)	528(19.1)	<0.001	660(62.1)	89(69.0)	571(61.2)	0.090
Rural (Lwak), n(%)	2475(79.1)	233(65.1)	2242(80.9)	Ref	402(37.9)	40(31.0)	362(38.8)	Ref
Household size								
<5, n(%)	1112(35.5)	126(35.2)	986(35.6)	0.880	443(41.7)	51(39.5)	392(42.0)	0.600
≥5, n(%)	2016(64.5)	232(64.8)	1784(64.4)	Ref	619(58.3)	78(60.5)	541(58.0)	Ref
Socioeconomic Status (Lwak only)								
1st quintile, n(%)	279(11.3)	26(11.2)	253(11.3)	Ref	42(10.4)	5(12.5)	37(10.2)	Ref
2nd quintile, n(%)	370(14.9)	34(14.6)	336(15.0)	0.960	61(15.2)	10(25.0)	51(14.1)	0.530
3rd quintile, n(%)	438(17.7)	49(21.0)	389(17.4)	0.430	68(16.9)	3(7.5)	65(18.0)	0.160
4th quintile, n(%)	473(19.1)	47(20.2)	426(19.0)	0.780	72(17.9)	4(10.0)	68(18.8)	0.230
5th quintile, n(%)	569(23.0)	50(21.5)	519(23.1)	0.800	71(17.7)	8(20.0)	63(17.4)	0.920
Unknown, n(%)	346(14.0)	27(11.6)	319(14.2)	0.500	88(21.9)	10(25.0)	78(21.5)	0.930

*chi-square test done only for those with malaria test

From the univariate analysis, few variables had p values <0.2: age, malaria, and urban/rural residence. These variables were included in multivariable analysis. For ILI, young age (0–5 months compared to 24–59 months, p = 0.01 and 6–23 months compared to 24–59 months, p<0.001), rural residence (p = 0.03) and positive malaria test (p<0.001) are negatively associated with influenza in both urban and rural areas when adjusted for age. For ALRI, malaria is only negatively associated with influenza in the rural, malaria-endemic area (Lwak) when adjusted for age (p = 0.048).

### Influenza-Associated Hospitalization Rates

Hospitalization was reported infrequently during home visits in Kibera (<1% of ALRI). The average rate of influenza-associated hospitalization was 4.3 (95% CI 3.0–6.0) per 1000 person-years in children aged 0–4 years in Lwak (Table 3). Hospitalization rates were highest in the 6–23 month age group at 8.4 (95% CI 5.4–13.0) and the 0–5 month age group at 7.6 (95% CI 3.2–18.2) per 1000 person-years. During the pandemic year, an exceptionally high hospitalization rate of 25.9 (95% CI 9.7–68.9) per 1000 person-years was detected among infants 0–5 months of age in Lwak. There were no differences in the median length of stay, proportion of cases with oxygen saturation <90%, or discharge diagnoses by age group among the 33 influenza-positive hospitalizations in Lwak (data not shown). Increased rates of influenza-associated hospitalizations during the 2009 pandemic resulted because of increases in total hospitalizations and a slight increase in the percent positive among tested patients compared to the mean over the study period (10% vs. 8.3%).

**Table 3:** Rate of Hospitalizations^a^ (Lwak) Attributable to Influenza per 1000 Person-Years, by Age Group and Year, 2008–2012.

Year	Unadjusted Rates				Adjusted Rates^a^			
	0–5 months	6–23 months	24–59 months	≤59 months	0–5 months	6–23 months	24–59 months	≤59 months
2008	0.0(-)	2.2(0.6–8.9)	1.2(0.3–4.7)	1.4(0.5–3.6)	0.0(-)	7.7(1.9–30.9)	4.7(1.2–19.0)	5.2(1.9–13.8)
2009	14.9(5.6–39.7)	4.2(1.6–11.1)	0.5(0.1–3.9)	2.9(1.5–5.6)	25.9(9.7–68.9)	8.7(3.3–23.1)	1.2(0.2–8.8)	6.1(3.2–11.7)
2010	3.7(0.5–26.0)	6.6(3.0–14.7)	1.0(0.3–4.1)	2.9(1.5–5.5)	4.7(0.7–33.5)	9.0(4.0–20.0)	1.5(0.4–5.9)	4.0(2.1–7.7)
2011	0.0(-)	4.9(1.8–13.0)	1.1(0.3–4.3)	2.1(0.9–4.6)	0.0(-)	5.9(2.2–15.7)	1.4(0.4–5.7)	2.6(1.2–5.9)
2012	0.0(-)	4.2(1.6–11.2)	0.5(0.1–3.5)	1.5(0.6–3.7)	0.0(-)	6.6(2.5–17.6)	1.1(0.2–7.7)	2.8(1.2–6.8)
**2008–2012**	**3.7(1.5–8.8)**	**4.4(2.8–6.8)**	**0.8(0.4–1.7)**	**2.2(1.5–3.0)**	**7.6(3.2–18.2)**	**8.4(5.4–13.0)**	**1.7(0.9–3.5)**	**4.3(3.0–6.0)**

^a^ Rates adjusted by applying the proportion influenza positive among hospitalized children meeting the acute lower respiratory tract illness (ALRI) case definition to all hospitalized children who met the ALRI case definition but did not have a laboratory result/sample collected and by applying the proportion of patients reporting hospitalization who were hospitalized at Lwak Mission Hospital

### Influenza-Associated Medically Attended ALRI Rates

During the 5 year study period, 55% of children in Lwak and 99% of children in Kibera meeting the ALRI case definition were treated as outpatients. The average rate of influenza-associated medically attended ALRI in children 0–4 years old was 17.4 (95% CI 14.2–19.7) per 1000 person-years in the two sites, 13.6 (95% CI 10.4–17.7) per 1000 person-years in Lwak and 20.2 (95% CI 16.4–24.9) per 1000 person-years in Kibera (Table 4). Rates of medically attended influenza-associated ALRI were highest in the 6–23 month age group at 29.6 (95% CI 22.9–36.1) per 1000 person-years. Rates of medically attended influenza-associated ALRI remained high among infants aged 0–5 months at 21.0 (95% CI 11.7–38.0) per 1000 person-years.

**Table 4:** Rate of Medically attended Acute Lower Respiratory Tract Illness (ALRI)^a^ Attributable to Influenza^b^ per 1000 Person-Years, by Site, Age Group and Year, Jan 2008—Dec 2012.

**Kibera**								
**Year**	**Unadjusted Rates** ^**a**^				**Adjusted Rates** ^**b**^			
	0–5 months	6–23 months	24–59 months	≤59 months	0–5 months	6–23 months	24–59 months	≤59 months
2008	4.4(0.6–31.3)	5.6(2.5–12.5)	3.1(1.5–6.6)	4.0(2.4–6.7)	71.6(10.1–508.2)	67.2(30.2–149.7)	25.2(12.0–52.8)	42.0(24.9–71.0)
2009	0.0(-)	16.8(11.2–25.4)	5.9(3.6–9.7)	8.8(6.4–12.1)	0.0(-)	104.7(69.6–157.6)	33.8(20.7–55.1)	54.9(40.1–75.1)
2010	13.9(5.8–33.3)	7.1(3.8–13.2)	3.5(1.9–6.4)	5.4(3.6–7.9)	50.0(20.8–120.2)	21.9(11.8–40.7)	10.7(5.7–19.8)	16.8(11.3–24.8)
2011	0.0(-)	3.1(1.1–8.1)	1.0(0.3–3.2)	1.6(0.7–3.3)	0.0(-)	9.6(3.6–25.5)	3.8(1.2–11.6)	5.4(2.6–11.4)
2012	0.0(-)	2.7(0.9–8.4)	0.4(0.1–2.8)	1.0(0.4–2.7)	0.0(-)	7.0(2.3–21.8)	1.6(0.2–11.1)	3.4(1.3–9.0)
**2008–2012**	**4.0(1.8–8.8)**	**7.3(5.5–9.8)**	**2.8(2.0–3.9)**	**4.2(3.4–5.2)**	**24.4(10.9–54.2)**	**34.5(25.9–46.1)**	**12.6(9.1–17.4)**	**20.2(16.4–24.9)**
**Lwak**								
**Year**	**Unadjusted Rates**				**Adjusted Rates** ^**b**^			
	**0–5 months**	**6–23 months**	**24–59 months**	**≤59 months**	**0–5 months**	**6–23 months**	**24–59 months**	**≤59 months**
2008	0.0(-)	2.2(0.6–8.9)	1.2(0.3–4.7)	1.4(0.5–3.6)	0.0(-)	18.2(4.6–72.8)	12.4(3.1–49.6)	12.9(4.9–34.4)
2009	14.9(5.6–39.7)	6.2(2.8–13.9)	2.2(0.8–5.8)	4.6(2.7–7.7)	53.1(19.9–141.5)	24.6(11.0–54.7)	9.6(3.6–25.5)	18.4(10.9–31.1)
2010	3.7(0.5–26.0)	8.8(4.4–17.6)	3.1(1.4–6.9)	4.8(2.9–8.0)	9.6(1.4–68.2)	19.6(9.8–39.2)	7.4(3.3–16.6)	11.2(6.7–18.5)
2011	0.0(-)	8.6(4.1–17.9)	1.6(0.5–5.0)	3.4(1.8–6.4)	0.0(-)	25.5(12.2–53.5)	4.3(1.4–13.3)	9.7(5.2–18.0)
2012	0.0(-)	5.2(2.2–12.6)	2.4(1.0–5.8)	3.1(1.6–5.7)	0.0(-)	16.4(6.8–39.3)	10.4(4.3–24.9)	11.5(6.2–21.5)
**2008–2012**	**3.7(1.5–8.8)**	**6.2(4.3–8.9)**	**2.1(1.4–3.3)**	**3.5(2.6–4.5)**	**17.2(7.2–41.3)**	**22.8(15.7–33.0)**	**8.5(5.5–13.1)**	**13.5(10.4–17.7)**
**Combined (Kibera and Lwak)**								
**Year**	**Unadjusted Rates**				**Adjusted Rates** ^**b**^			
	**0–5 months**	**6–23 months**	**24–59 months**	**≤59 months**	**0–5 months**	**6–23 months**	**24–59 months**	**≤59 months**
2008	1.8(0.3–13.0)	4.1(2.0–8.2)	2.3(1.2–4.4)	2.8(1.8–4.4)	29.6(3.9–198.6)	44.8(22.7–90.7)	19.6(13.0–47.9)	28.8(20.4–51.3)
2009	6.4(2.4–17.0)	12.5(8.7–17.9)	4.4(2.8–6.8)	7.1(5.4–9.3)	22.8(13.5–96.1)	71.6(43.9–90.9)	24.0(13.7–33.0)	39.9(27.2–46.6)
2010	9.5(4.3–21.1)	7.8(4.9–12.3)	3.3(2.0–5.4)	5.1(3.8–7.0)	32.6(14.5–72.0)	21.0(13.3–33.6)	9.4(5.5–14.5)	14.5(10.5–19.4)
2011	0.0(-)	5.2(2.9–9.3)	1.3(0.6–2.8)	2.3(1.4–3.7)	0.0(-)	15.7(8.8–28.5)	4.0(1.7–8.6)	7.1(4.5–11.6)
2012	0.0(-)	3.9(1.9–7.7)	1.3(0.6–2.9)	2.0(1.2–3.3)	0.0(-)	11.3(5.8–23.2)	5.5(2.3–11.6)	7.1(4.1–11.7)
**2008–2012**	**3.8(2.1–6.9)**	**6.9(5.5–8.6)**	**2.5(1.9–3.3)**	**3.9(3.3–4.6)**	**21.0(11.7–38.0)**	**29.6(22.9–36.1)**	**10.9(7.9–13.3)**	**17.4(14.2–19.7)**

^a^ Includes hospitalized and out-patient children who met the ALRI case definition: Cough or difficulty breathing AND a danger sign (child unable to drink or breastfeed, child vomits everything, child had convulsions, child is lethargic or unconscious) or oxygen saturation <90%;

^b^ Rates adjusted by applying the proportion influenza positive among hospitalized and outpatient children meeting the ALRI case definition to all hospitalized and out-patient children who met the ALRI case definition but did not have a laboratory result/sample collected

There was no difference between influenza-positive inpatients and influenza-positive ALRI outpatients in gender or age distribution (Table 5). Outpatient influenza-associated ALRI case-patients in Lwak and Kibera were as likely as hospitalized influenza-associated case-patients to be hypoxic (p = 0.50). Hospitalized case-patients with influenza were more likely to be diagnosed with malaria (p = <0.001). Convulsions were more likely to be reported among non-hospitalized ALRI cases and hospitalized cases in Lwak, than among non-hospitalized ALRI cases in Kibera (p<0.001). Chest wall indrawing was more frequently reported among non-hospitalized ALRI cases in Kibera than among hospitalized cases or non-hospitalized ALRI cases in Lwak (p<0.001).

**Table 5:** Characteristics of Hospitalized (Lwak only) and Medically attended Non-Hospitalized Acute Lower Respiratory Tract Illness (ALRI)^a^ (Lwak and Kibera) Attributable to Laboratory-Confirmed Influenza, 2008–2012.

	Hospitalized ALRI (Lwak) (N = 33)	Non-Hospitalized ALRI (Lwak)^a^ (N = 20)	Non-Hospitalized ALRI (Kibera)^a^ (N = 89)	p-value^b^
	n (%)	n (%)	n (%)	
Sex				
Male	18(54.5)	11(55.0)	50(56.2)	0.985
Age				
0–5 months	5(15.2)	0(0.0)	6(6.7)	0.161
0–11months	12(36.4)	4(20.0)	25(28.1)	0.422
6–23 months	20(60.6)	8(40.0)	46(51.7)	0.348
12–23 months	13(39.4)	4(20.0)	27(30.3)	0.351
24–59 months	8(24.2)	12(60.0)	37(41.6)	0.031
Oxygen Saturation <90%	2(6.1)	4(20.0)	10(11.2)	0.288
Unable to drink/drinks poorly	1(3.0)	2(10.0)	2(2.2)	0.222
Vomits everything	4(12.1)	3(15.0)	20(22.5)	0.45
Had convulsions	6(18.2)	6(30.0)	1(1.1)	<0.001
Was lethargic	6(18.2)	4(20.0)	5(5.6)	0.032
Had chest wall in-drawing	9(27.3)	0(0.0)	57(64.0)	<0.001
Had stridor	3(9.1)	0(0.0)	2(2.2)	0.122
Diagnosis—n (%)				
Influenza	0(0.0)	1(5.0)	0(0.0)	0.377
Pneumonia/Lower Respiratory Tract Infection	15(45.5)	2(10.0)	58(65.2)	<0.001
Malaria	19(57.6)	7(35.0)	9(10.1)	<0.001
Tuberculosis	1(3.0)	1(5.0)	0(0.0)	0.138
Other	10(30.3)	12(60.0)	42(47.2)	0.085

^a^ Includes out-patient children who met the ALRI case definition: Cough or difficulty breathing AND a danger sign (child unable to drink or breastfeed, child vomits everything, child had convulsions, child is lethargic or unconscious) or oxygen saturation <90%

^b^ Fisher's exact.

## Discussion

Rates of influenza-associated hospitalizations in Kenyan children aged 0–4 years were higher than U.S. estimates in 2008 before the 2009 influenza A(H1N1) pandemic and continued to be higher than U.S. estimates during and after the pandemic period. The prolonged circulation of influenza throughout most of the year and higher prevalence of co-morbid conditions (e.g. HIV, TB, malaria, malnutrition) may contribute to these rates. Prior studies have documented the association of HIV and increased severity (hospitalizations, deaths) of influenza virus infections.[12, 13, 27, 28] Likewise, tuberculosis has been associated with increased severity of influenza-associated illness.[29, 30] Data on the interaction of influenza and malaria or malnutrition have been mixed and warrant further study.[31–37]

Influenza was rarely clinically diagnosed among children with severe laboratory-confirmed influenza by inpatient or outpatient care providers, which implies poor recognition of influenza as a respiratory pathogen. A study of U.S. children with laboratory-confirmed influenza during 2000–2004 found that only 28% of inpatients and 17% outpatients with laboratory-confirmed influenza were clinically diagnosed with influenza by their healthcare provider.[38] In this study, children aged 6–23 months had the highest rates of influenza-associated hospitalization, which may reflect actual increased burden or incomplete detection in infants aged 0–5 months. Likewise, although medically attended outpatient ALRI episodes appeared to have similar severity (as determined by the presence of hypoxia and danger signs) to inpatient episodes, few children in the urban setting were hospitalized with influenza-associated ALRI.

From 2008–2012, annual influenza-associated hospitalization rates for Kenyan children aged 0–4 years were 5–10 times higher than estimates in U.S. children by the Centers for Disease Control and Prevention’s (CDC’s) Emerging Infections Program (EIP). EIP estimates ranged from 0.2–0.3 hospitalizations per 1000 children 0–4 years old in non-pandemic years to 0.8 per 1000 during the 2009–10 pandemic.[39, 40] Surveillance during the 2009 influenza A(H1N1) pandemic in the United States found that infants aged 0–5 months had the highest hospitalization rate at 2.0 per 1000, followed by children aged 6–23 months with a rate of 0.9 per 1000.[41] In our study in Kenya, rates of hospitalization during the 2009 A(H1N1) pandemic were 6–12 times U.S. EIP estimates for the same age strata.[41] In a recent publication that used an alternative methodology for calculating influenza-associated hospitalization rates in Kenyan children during 2009–2012 estimated an average rate of 2.7 (95% CI 1.8–3.9) per 1000 children aged 0–4 years: 5.7(95% CI 2.4–13.8) per 1000 children aged 0–5 months; 4.7(95% CI 1.8–11.9) per 1000 children aged 6–11 months; 4.4(95% CI 2.3–8.5) per 1000 children aged 12–23 months, and 1.4 (95% CI 0.7–2.7) per 1000 children aged 24–59 months.[42] These data demonstrate the unusually high burden of severe disease due to seasonal and pandemic influenza in young Kenyan children. A recent publication from South Africa reports 7% of ALRI hospitalizations among children aged 0–4 years during 2009–2012 were influenza-associated [28]. Applying 7% positivity to the annual rates of ALRI hospitalization gives an annual rate of 1.8–2.2 per 1000 children during 2009–2012 (though likely there is greater year to year fluctuation in percent positive). These estimates fall within the confidence limits of our own annual estimates for all but the pandemic year, but the confidence intervals for the pandemic year still overlap.

Children with influenza-associated outpatient ALRI were equally as likely to be hypoxic as inpatients with influenza infection suggesting similar levels of disease severity; therefore, we conducted additional analyses including all children who met the ALRI case definition and sought medical care to estimate the prevalence of severe influenza-associated illness. Using this method, rates of severe disease (influenza-associated hospitalization and non-hospitalized medically attended influenza-associated ALRI) were approximately 4 times greater than hospitalization rates alone.

An earlier publication estimated the incidence of influenza-associated SARI during 2007–2010 in Lwak in children 0–4 years old to be 58 per 1000 person-years of surveillance.[19] This rate is much higher than our medically attended ALRI estimate presumably because it includes the pandemic year, 2 non-pandemic years and non-medically attended SARI. Our estimates of influenza-associated medically attended ALRI in the 2009 pandemic were approximately 3 times higher than non-pandemic years for children aged 6–23 months and children aged 24–59 months. Additional years of surveillance outside the pandemic period may provide greater stability to these estimates.

There were several limitations to this study. Influenza was rarely identified in children 0–5 months old with hospitalized ALRI. The lowest rates of medically attended ILI were also among children 0–5 months of age which may reflect a greater tendency to hospitalize in this age group, or atypical presentation of influenza in young infants. A study of 401 children hospitalized with laboratory-confirmed influenza from 1988–2004 in Finland found that over half of children 0–5 months old were admitted for suspected sepsis, and nearly half of children 6 months-2 years old and over two-thirds of children 3–6 years old were admitted with non-respiratory primary diagnoses.[43] This suggests case definitions for young infants might need to be broadened to include hypothermia or fever alone or other respiratory symptoms to improve influenza detection. To support this, higher rates of influenza-associated hospitalizations were found among infants aged 0–5 months at Siaya District Hospital where presence of fever was not required to meet the SARI case definition.[42] In Kenya, there are many barriers preventing hospitalization of ill children. Many parents cannot afford transport to health facilities or to pay for hospitalization charges, parents may be unable to stay in the hospital to care for young children because they need to work or care for other members of the household, and hospitalization and oxygen administration are frequently associated with death in young children making parents reluctant to seek hospital-based care for a severely ill infant.[44] The other criteria that distinguish outpatient ALRI from ILI is the presence of one or more danger signs. Some danger signs may be misinterpreted by clinicians or caregivers leading to over estimation of severe disease in children. In this study, the increased reporting of chest wall indrawing may reflect differences in clinical practice at the two sites rather than a real difference in the frequency of respiratory distress but this is difficult to determine without a gold standard physical examination. The higher rate of convulsions reported in the rural area may be partially attributable to the co-circulation of malaria but may also reflect differences in clinical practice at the two study sites.

Finally, this study assumed that rates of influenza illness in children with ILI or ALRI who were not tested for influenza were similar to rates of influenza among children with ILI or ALRI who were tested for influenza. Severely ill children were often excluded from enrolment because of clinical instability, which may bias our estimates if influenza was more or less common among these children than enrolled children. Likewise, influenza-associated illness may have been more or less severe than other causes of ILI leading to differential care-seeking that could bias the proportion positive.

The burden of influenza-associated hospitalization in Kenyan children from 2008–2012 was at least 5–10 times higher than contemporaneous U.S. estimates.[45] Many children who had an IMCI danger sign were not hospitalized which may indicate that the true burden of influenza-associated severe disease in Kenyan children is higher than current estimates suggest. Influenza-associated hospitalizations in infants aged 0–5 months were likely underestimated in our surveillance, due to the atypical presentation of influenza in this age group. Few clinicians diagnosed children with influenza despite the presence of a global pandemic during the reporting period. Influenza-associated disease remains under-recognized in Kenyan children. In research settings for burden of disease estimates, expanding case definitions for children aged 0–5 months to include history of fever or hypothermia without respiratory symptoms may increase sensitivity of influenza detection. Expanding case definitions in children 6–59 months of age to include fever and cough in children without a primary respiratory diagnosis may also increase sensitivity of influenza detection. Furthermore, clinicians should be sensitized to recognize influenza as an important cause of severe disease in young children. Influenza vaccination should be strongly considered.

## Appendix 1

### Equations used in calculation of incidence rates of influenza-associated acute lower respiratory tract illness (ALRI) hospitalizations and outpatient visits

#### Equation 1a: Incidence rate of influenza-associated hospitalization

$$IR_{hosp}=\frac{HospFlu_{cases}}{PoY}$$

Where:

*IR*_*hosp*_ = Unadjusted incidence rate of influenza-associated ALRI hospitalizations

*HospFlu*_*cases*_ = Total number of hospitalized ALRI cases who tested positive for influenza

*PoY* = Person-time of surveillance in years

#### Equation 1b: Incidence rate of influenza-associated medically attended ALRI

$$IR_{medALRI}=\frac{MedFlu_{cases}}{PoY}$$

Where:

*IR*_*medALRI*_ = Unadjusted incidence rate of influenza-associated medically attended ALRI (in- and outpatients)

*MedFlu*_*cases*_ = Total number of medically attended ALRI cases (in- and outpatients) who tested positive for influenza

*PoY* = Person-time of surveillance in years

#### Equation 2a: Adjusted incidence rate of influenza-associated hospitalization

$$AdjIR_{hosp}=\left(IR_{hosp}\times \frac{1}{P_{hospALRI}}\times \frac{1}{P_{hospflutest}}\right)$$

Where:

*AdjIR*_*hosp*_ = Adjusted incidence rate of influenza-associated hospitalized ALRI

*IR*_*hosp*_ = Unadjusted incidence rate of influenza-associated hospitalizations

*P*_*hospALRI*_ = Proportion of cases in the community who met the ALRI case definition and were hospitalized at the study hospital

*P*_*hospflutest*_ = Proportion of hospitalized patients who met the ALRI case definition and were tested for influenza

#### Equation 2b: Adjusted incidence rate of influenza-associated medically attended ALRI

$$AdjIR_{medALRI}=\left(IR_{medALRI}\times \frac{1}{P_{medALRIflutest}}\right)$$

Where:

*AdjIR*_*medALRI*_ = Adjusted incidence rate of influenza-associated medically attended ALRI (in- and outpatients)

*IR*_*medALRI*_ = Unadjusted incidence rate of influenza-associated medically attended ALRI (in- and outpatients)

*P*_*medALRIflutest*_ = Proportion of in- and outpatients who met the ALRI case definition and were tested for influenza

## Supporting Information

S1 Table Person-time of Surveillance in weeks in Lwak and Kibera for Children 0–4 years of Age by Calendar Month, 2008–2012. (DOCX)
