# Supplementary material for: The Unrecognized Burden of Influenza in Young Kenyan Children, 2008-2012
Source: PLoS One. 2015 Sep 17;10(9):e0138272. doi: 10.1371/journal.pone.0138272 (PMC4574572; doi:10.1371/journal.pone.0138272)
Supplement: S1 Table — (DOCX) [file pone.0138272.s001.docx]

**S1 Table. Person-time of Surveillance in weeks in Lwak and Kibera for Children 0-4 years of Age by Calendar Month, 2008-2012.**

| **Year** | **Month** | **Kibera** | **Lwak** | **Total** |
| --- | --- | --- | --- | --- |
| **2008-12** | **Jan** | 1604.8 | 1159.6 | 2764.4 |
|  | **Feb** | 1645.1 | 1254.8 | 2899.9 |
|  | **Mar** | 1882.5 | 1413.2 | 3295.7 |
|  | **Apr** | 1873.2 | 1394.9 | 3268.1 |
|  | **May** | 1944.6 | 1420.7 | 3365.2 |
|  | **Jun** | 1826.4 | 1402.4 | 3228.9 |
|  | **Jul** | 1923.3 | 1379.4 | 3302.7 |
|  | **Aug** | 1923.5 | 1409.7 | 3333.2 |
|  | **Sep** | 1958.0 | 1401.6 | 3359.6 |
|  | **Oct** | 1782.6 | 1369.2 | 3151.8 |
|  | **Nov** | 1776.8 | 1278.0 | 3054.8 |
|  | **Dec** | 878.4 | 431.2 | 1309.7 |
